# Graphene Plasmonic Metasurfaces to Steer Infrared Light

**DOI:** 10.1038/srep12423

**Published:** 2015-07-23

**Authors:** Zubin Li, Kan Yao, Fengnian Xia, Sheng Shen, Jianguo Tian, Yongmin Liu

**Affiliations:** 1 Department of Mechanical and Industrial Engineering, Northeastern University, Boston, Massachusetts 02115, United States; 2 Key Laboratory of Weak-Light Nonlinear Photonics, Ministry of Education, TEDA Applied Physics Institute & School of Physics, Nankai University, Tianjin 300457, China; 3 Department of Electrical and Computer Engineering, Northeastern University, Boston, Massachusetts 02115, United States; 4 Department of Electrical Engineering, Yale University, New Haven, Connecticut 06511, United States; 5 Department of Mechanical Engineering, Carnegie Mellon University, Pittsburgh, Pennsylvania 15213, United States

## Abstract

Metasurfaces utilizing engineered metallic nanostructures have recently emerged as an important means to manipulate the propagation of light waves in a prescribed manner. However, conventional metallic metasurfaces mainly efficiently work in the visible and near-infrared regime, and lack sufficient tunability. In this work, combining the pronounced plasmonic resonance of patterned graphene structures with a subwavelength-thick optical cavity, we propose and demonstrate novel graphene metasurfaces that manifest the potential to dynamically control the phase and amplitude of infrared light with very high efficiency. It is shown that the phase of the infrared light reflected from a simple graphene ribbon metasurface can span over almost the entire 2π range by changing the width of the graphene ribbons, while the amplitude of the reflection can be maintained at high values without significant variations. We successfully realize anomalous reflection, reflective focusing lenses, and non-diffracting Airy beams based on graphene metasurfaces. Our results open up a new paradigm of highly integrated photonic platforms for dynamic beam shaping and adaptive optics in the crucial infrared wavelength range.

Metamaterials are rationally engineered composite materials with exotic properties not easily obtained or altogether unavailable in nature^1^,^2^,^3^,^4^. Metamaterials have attracted extensive attention from physicists, material scientists and engineers over the past decade, because they provide unprecedented opportunities to control light-matter interactions and light propagation in unusual ways. Recently, metasurfaces, a new class of two-dimensional (2D) metamaterials, have emerged at the frontier of metamaterials research^5^,^6^,^7^. In essence, metasurfaces introduce desired phase, amplitude, or polarizatin profiles by patterning planar subwavelength structures, offering additional, yet important degrees of freedom to mold the flow of light. Novel optical phenomena and devices based on metasurfaces include anomalous refraction or reflection^8^,^9^, strong spin-orbit interaction^10^,^11^, ultra-thin focusing or diverging lenses^12^,^13^,^14^, and holography^15^,^16^. Metasurfaces have also been applied to manipulate near-field surface waves^17^,^18^,^19^,^20^. Compared with their bulk metamaterial counterparts, the quasi-2D metasurfaces exhibit advantages of increased operation bandwidth and reduced losses^5^,^6^. These new designs are also compatible with planar, low-cost manufacturing and extremely suitable for device integration.

So far, most metasurfaces rely on judiciously designed metallic nanostructures. At optical frequencies, metallic nanostructures with varied geometries support strong surface plasmon resonances, acting as local antennas in frequency selective surfaces (FSS)^21^ that can eventually determine the direction, amplitude and polarization of refracted or reflected beams. However, it is known that the plasmonic response of metals becomes less pronounced as approaching mid-infrared or longer wavelengths because of the weaker interaction between electromagnetic waves and electrons^22^,^23^,^24^. This limits the use of noble metals in many infrared applications. Graphene, a monolayer of carbon atoms arranged in a honeycomb lattice^25^,^26^, has emerged as a promising, alternative candidate for plasmonics at terahertz and mid-infrared frequencies^27^,^28^,^29^,^30^,^31^,^32^,^33^,^34^. Graphene’s electron density, and consequently its plasma frequency, is much lower than that of bulk noble metals. As a result, graphene is able to support surface plasmons as a bulk metal does, whereas operating at mid-infrared and lower frequencies. Because of the 2D nature of the platform, graphene surface plasmons exhibit extremely small wavelengths (~λ/10–λ/100) and tight field confinement on the graphene sheet^33^,^34^, while maintaining reasonably small losses in the terahertz and mid-infrared regime. Indeed, they closely resemble plasmons in the two-dimensional electron gases (2DEGs) that have been explored before^35^,^36^,^37^. The strong optical response arising from graphene surface plasmons also allows us to build novel metamaterials^38^,^39^,^40^. Moreover, the mechanical, electronic, optical, and even thermal properties of graphene are highly tunable via chemical doping or electrical gating^41^,^42^,^43^,^44^,^45^,^46^,^47^, which is impossible or inefficient if metals are used. These favorable features arouse enormous interest in the investigation of graphene-based tunable plasmonics and metamaterials^31^,^32^,^38^,^39^,^40^,^48^,^49^.

Although fairly extensive effort has been devoted to graphene-based metamaterials and plasmonics, previous work mainly focuses on the distinct resonance features and their spectral tuning. In this letter, we propose and demonstrate graphene plasmonic metasurfaces to steer infrared light with high efficiency, which drastically expand the functionalities and performance of graphene devices towards complete wave shaping. This is made possible by combining the plasmonic resonance of patterned graphene nanostructures with a subwavelength-thick optical cavity to enhance the intrinsically weak interaction between light and single-layer graphene. Furthermore, the graphene metasurfaces can be dynamically tuned by changing the Fermi energy of graphene via simple chemical or electrostatic tuning that is unachievable in conventional metasurfaces made of metals. As a proof of principle, we numerically study graphene metasurfaces consisting of rationally designed graphene ribbons. It is shown that the phase of the reflected infrared light can range almost from –π to π by tailoring the dimensions of the graphene ribbons as well as the optical cavity, while the amplitude of the reflectivity is sufficiently high without substantial variations. This allows us to design graphene metasurfaces to implement anomalous reflection, focusing and non-diffracting Airy beam generation. We envision that such transformative graphene metasurfaces would open up a new paradigm of integrated photonic systems with multifunctional applications, including dynamic beam shaping, hyperspectral imaging, adaptive optics, biomedical sensing, and energy harvesting in the critical infrared region.

## Results

**Figure 1:**
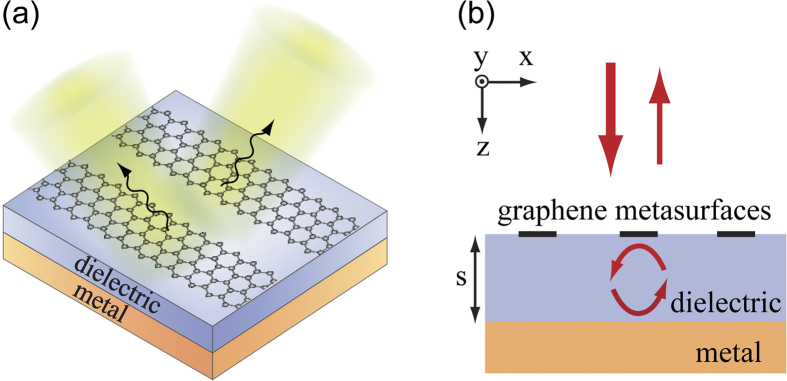
(**a**) Schematic of graphene plasmonic metasurfaces, which consist of subwavelength patterned graphene ribbons on a dielectric/metal substrate. By changing the geometry of the graphene ribbons and the thickness of the dielectric layer, the resultant plasmonic resonance varies and the reflected infrared light can be controlled in a prescribed manner. The incident light is not shown for clarity. (**b**) The graphene plasmonic metasurface forms an asymmetric Fabry-Perot resonator to substantially enhance the interaction between light and monolayer graphene structures.

Graphene can interact with light strongly via plasmonic resonance^27^,^38^,^39^,^50^,^51^. However, due to its innate thinness, light-graphene interactions need to be further enhanced to achieve adequate efficiency for practical device applications. Towards this goal, we utilize a subwavelength-thick optical cavity, which is composed of patterned graphene nanostructures, a dielectric spacing layer and an optically thick metal film as shown in Fig. 1a. This structure can be modeled as an asymmetric Fabry-Perot resonator, that is, a metasurface reflector as a partially reflecting mirror in the front and a metallic fully reflecting mirror in the back. When a plane wave is incident on the metasurface, the reflected fields from each interface interfere with each other (Fig. 1b). Depending on the geometry of the graphene nanostructures, and the dielectric constant and thickness of the dielectric layer, critical coupling condition can be satisfied even when the thickness *s* of the dielectric spacing layer is smaller than the wavelength. Consequently, we can achieve strong and even perfect absorption with monolayer graphene structures as theoretically proposed and recently demonstrated in experiments^52^,^53^,^54^,^55^.

Graphene metasurfaces coupled with an optical cavity exhibit much more striking properties and applications beyond strong absorption. Our numerical simulations show that the phase of the reflected light can vary from almost –π to π by selecting the width of the graphene ribbons as well as the thickness of the dielectric spacing layer, while the amplitude of reflectivity remains high and only shows moderate variations. These two properties allow us to design a variety of planar graphene photonic devices in the reflective configuration that are easy to fabricate and integrate. It should be emphasized that the full 2π phase coverage is crucial for designing functional metasurfaces, which necessitates the bottom metallic mirror. The bare graphene ribbons on a dielectric layer only provide a phase modulation range of about π (Supplementary Information)^56^, because of its intrinsic Lorenz-like resonance. To cover the full phase space, one can utilize V-shape antennas or geometric phase by rotating the structures. However, these two approaches only work for cross-polarization, and the efficiency is generally low^8^,^9^,^13^,^57^.

**Figure 2:**
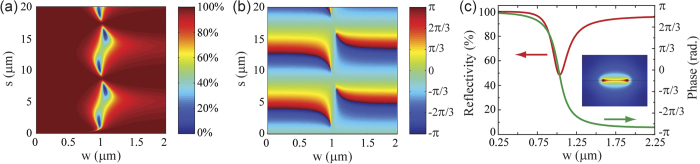
(**a**) Reflectivity and (**b**) relative phase of the reflected light as the functions of ribbon width (*w*) and dielectric layer thickness (*s*). (**c**) Reflectivity and phase of the reflected light with varied ribbon widths, while the thickness of the dielectric layer is fixed at *s *= 4.37 μm. The inset shows the field distribution at the cross-section of a graphene ribbon at the plasmonic resonance condition when *w *= 1.035 μm. The frequency of infrared light is 12.32 THz, the refractive index of the dielectric layer is 1.4, and the Fermi energy of graphene is 0.64 eV in all simulations.

In this paper, we focus on the far-infrared region, while the operation wavelength of the proposed graphene metasurfaces can be readily extended to mid-infrared by scaling the dimension of the graphene patterns. Figure 2a,b show the reflectivity and the phase of the reflected light with transverse magnetic (TM) polarization, respectively, as the function of the thickness (*s*) of the dielectric spacing layer and the width (*w*) of the graphene ribbons. TM polarized light can excite the plasmonic resonance of graphene ribbons. In contrast, for transverse electric (TE) polarization, the electric field is parallel to the graphene ribbons, and no plasmonic resonance can be excited to modulate the phase and amplitude of reflected light (Supplementary Information). The frequency of the infrared light is 12.32 THz, which is the resonant frequency for a graphene ribbon with width 1.035 μm. The periodicity (*p*) of the graphene ribbon is fixed at 3 μm. In the finite element simulation utilizing commercial software COMSOL Multiphysics, the optical conductivity of graphene is described by Kubo model with the Fermi energy *E*_*f*_ = 0.64 eV. The refractive index of the dielectric spacing layer is taken as 1.4, and Drude model is applied to fit the experimental data of permittivity of gold for the back reflective mirror^58^. More details about the simulation can be found in the Methods session.

From Fig. 2a,b, one can clearly see that there is a pronounced resonance feature when the width of the graphene ribbons is about 1 μm. The reflection amplitude and phase vary drastically around this value. Meanwhile, if we change the substrate thickness, the reflection and the phase show periodic oscillations. At certain thicknesses, the reflection can drop to almost zero when the width of the graphene ribbons is within the resonance region. This means that the absorption approaches unity, since the gold back mirror is optically thick and there is no transmission. Such perfect absorption is consistent with previous published work^52^,^53^. At other thicknesses, for example, *s *= 4.37 μm, the reflectivity is larger than 48.6% and the phase smoothly spans over from almost –π to π with different graphene ribbon widths as shown in Fig. 2c. The finite loss of graphene limits the phase shift range, which is known for a damped oscillator. In the ideal, lossless case, the phase shift can completely cover the 2π range. The inset of Fig. 2c plots the amplitude of the electric field within one unit cell of the graphene metasurface at the plasmonic resonance frequency. It can be seen that the field is extremely confined in the vicinity of the graphene ribbon, implying negligible interactions between adjacent unit cells. Therefore, the phase diagram calculated from periodic graphene ribbons well represents the intrinsic resonance of individual ribbons rather than their collective behavior.

The results in Fig. 2c indicate that we can achieve high reflectivity larger than 48.6% and a continuous 2π phase modulation by changing the width of the graphene ribbons, enabling design of novel planar optical devices to steer reflected infrared light with high efficiency. We start with the demonstration of anomalous reflection. In metasurfaces, new degrees of freedom to control light propagation are attained by introducing in-plane phase gradient *dφ/dx* at the interface between two media. Anomalous reflection and refraction phenomena have been reported in this regime, which are in sharp contrast to the well-known refraction and reflection law^8^,^9^. For the latter case, the relationship between the reflection angle and incident angle is generalized to


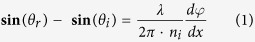


where *n*_*i*_ is the refractive index of the material at the incidence side, and *θ*_*i*_ and *θ*_*r*_ are the incident and reflected angle, respectively. There is a nonlinear relation between *θ*_*i*_ and *θ*_*r*_, distinctly different from conventional specular reflection. For normal incidence and *n*_*i*_ = 1 (air), the reflection angle can be calculated as


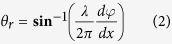


**Figure 3:**
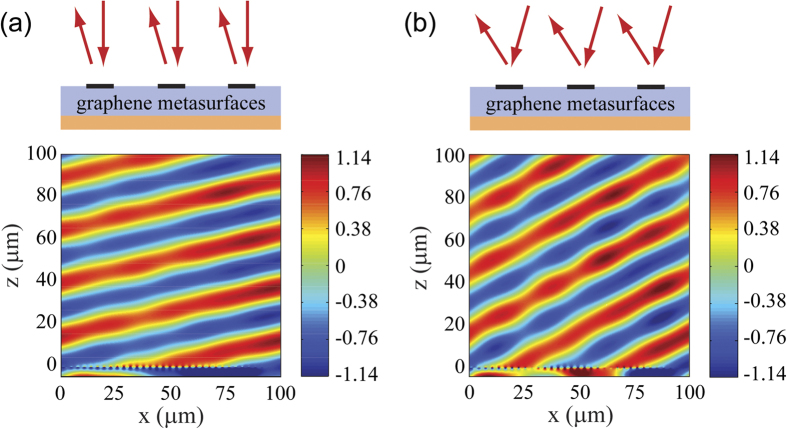
Demonstration of anomalous reflection for (**a**) normal incidence, and (**b**) oblique incidence with the incident angle of 15.0°. In the figures, the incident field has been subtracted, and the plotted *H*_*y*_ field component of the reflected light is normalized to the incidence. The frequency of infrared light is 12.32 THz and the Fermi energy of graphene is taken as 0.64 eV. The refractive index of the dielectric spacing layer is 1.4, and its thickness is 4.37 μm. Graphene ribbons are located at the plane of *z* = 0.

In our simulation, we set 36 graphene ribbons with periodicity of 3 μm. The width of each ribbon is properly selected (Supplementary Information) so that there is 10° phase shift between two adjacent unit cells. For normally incident light at 12.32 THz, we can deduce that the reflection angle is 13.0° from the phase front. The full-wave simulation result is shown in Fig. 3a, which plots the magnetic field (*H*_*y*_) of the reflected light. According to the field distribution, we can retrieve the reflection angle, which is about 13.1° and in excellent agreement with the predicted value. The slight deformation of the phase front arises from the finite periodicity and non-uniform reflectivity of the metasurface. If we set the incident angle as 15.0°, the reflected angle should be about 29.0°. The simulation result is shown in Fig. 3b, and we can see that the reflected light is again like a plane wave with its phase front tilted about 28.9° relative to the surface normal. The overall reflectivity is about 73.9% for normal incidence and 76.0% for 15.0° incidence, clearly demonstrating the high efficiency of the graphene metasurface.

The second graphene metasurface device to be demonstrated is a reflective focusing lens. To design such a lens, we must ensure that the light reflected from every graphene ribbon has the same phase or with 2π multiplied by an arbitrary integer when propagating to the focal point. In other words, the metasurface should carry a phase profile to compensate the propagation phase between graphene ribbons and the focal point, so that the radiated waves from the metasurface can constructively interfere with each other at the focal point. For a certain focal length *F*, the phase shift distribution *φ*(x) along the interface should satisfy


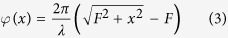


**Figure 4:**
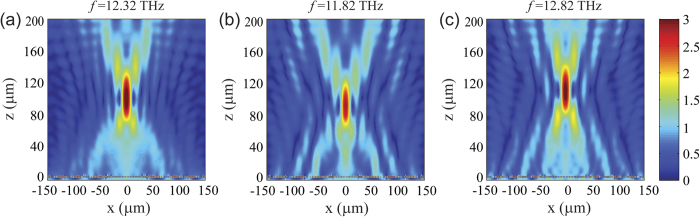
Demonstration of reflective focusing lens at three different frequencies : (**a**) 12.32 THz, (**b**) 11.82 THz, and (**c**) 12.82 THz. In the figures, the incident light has been subtracted, and the amplitude of the reflected light is normalized to the incidence. Material parameters are the same as in Fig. 3. Graphene ribbons are located at the plane of *z *= 0.

In our study, we set *F* = 100 μm, and *x* = *mp* with *p* equal to the periodicity of the graphene ribbons and *m* = 0, ±1, ±2… From the results presented in Fig. 2c, we can determine the width of graphene ribbons along the *x* axis (Supplementary Information) to match the phase profile calculated by Equation 3. The simulation result is shown in Fig. 4a. TM polarized light at frequency *f* = 12.32 THz is launched from the top, and perpendicularly impinges upon the graphene metasurface. One can see clearly that the reflection is efficiently focused. The focal point is at 98.8 μm, agreeing very well with the original design at 100 μm. The small discrepancy between the design and simulation is mainly attributed to the finite size of the unit cells with respect to the wavelength. In addition, the focusing effect of the graphene metasurface shows reasonable operation bandwidth. Figures 4b,c present the simulation results at 11.82 and 12.82 THz, respectively. The focusing effect is still preserved, although the field intensity at the focal point slightly decreases and the focal point shifts about ±10 μm compared to Fig. 4a.

**Figure 5:**
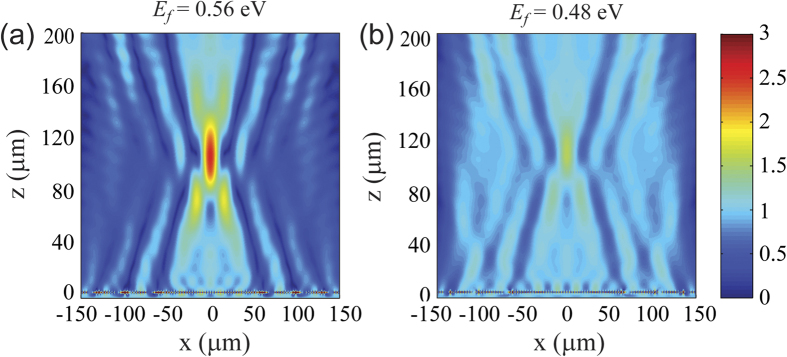
Dynamically tunable reflective focusing lens by varying the Fermi energy of graphene. (**a**) *E*_*f*_ = 0.56 eV and (**b**) *E*_*f*_ = 0.48 eV. In the figures, the incident light has been subtracted, and the amplitude of the reflected light is normalized to the incidence. The operation frequency is 12.32 THz. Graphene ribbons are located at the plane of *z* = 0.

The optical property of graphene strongly depends on its carrier concentration, and hence the Fermi energy of the graphene sheet. This allows us to realize dynamically tunable metasurfaces, which are impossible or inefficient if metallic nanostructures are employed. In Fig. 5, we compare the same graphene focusing metasurface at the same operation frequency of 12.32 THz while reducing the Fermi energy of graphene from 0.64 eV used in Fig. 4a to 0.56 eV and 0.48 eV, respectively. It can be seen that the focal point shifts slightly to a longer distance, and the intensity at the focal point significantly reduces. The focusing effect can be completely turned off when the Fermi energy is about 0.4 eV or smaller. If we increase the Fermi energy to a value larger than 0.64 eV, similar strong modulation effect is observed while the focal point slightly moves towards the metasurface. These results manifest a new avenue for dynamic beam shaping and adaptive optics on a truly 2D platform through simple electrostatic gating.

**Figure 6:**
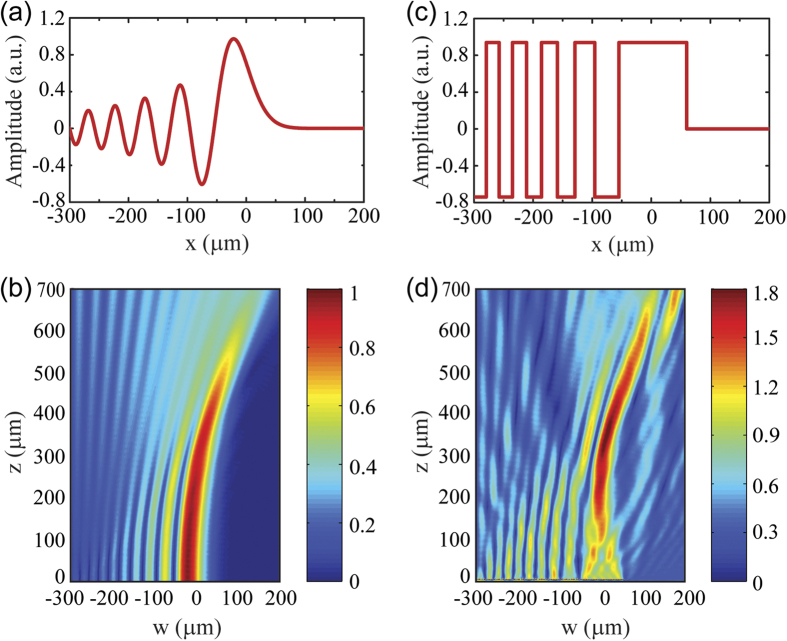
Generation of non-diffracting Airy beam. (**a**) Ideal phase and amplitude at the emitting interface to generate an Airy beam. (**b**) Electric filed amplitude of an ideal airy beam. (**c**) Simplified Airy function. (**d**) An Airy beam generated by a graphene metasurface following the simplified design.

Finally we employ graphene metasurfaces to generate Airy beams. Airy beams represent the only possible non-diffracting wave packets in one-dimensional (1D) planar systems^59^,^60^. In addition to their non-diffracting and self-healing properties^61^, the propagation of Airy beams exhibits a unique self-bending behavior in the absence of any external potential, which has been exploited for interesting applications in optical micromanipulation^62^. Airy beams can be generated by a designed phase and amplitude profile at the emitting plane, which is given by Airy function (Fig. 6a). The field plot of the resulting ideal Airy beam is presented in Fig. 6b, showing clearly the exceptional self-bending and non-diffractive feature. However, the ideal phase and amplitude profile cannot be easily satisfied with graphene ribbons, because it is very difficult to modulate the phase and the amplitude separately by just changing the width and periodicity of the ribbons. Fortunately, a simplified yet practical approach is to just consider the phase of the Airy function and keep the emitting field amplitude constant^63^. The field amplitude modulation is not so crucial because of Airy beam’s special self-healing property, which can automatically correct the field profile along the propagation path even in the presence of defects. The peaks and valleys in Fig. 6a can be approximated as rectangular profiles with equal magnitudes and a π phase difference as shown in Fig. 6c. In our simulation, we use two ribbon widths, *w*_1_ = 858 nm (for peaks) and *w*_2_ = 1058 nm (for valleys) to match the simplified profile (Supplementary Information). From Fig. 6d one can see that under such an approximation, the reflected field still well resembles the ideal Airy beam shown in Fig. 6b.

## Discussion

We have introduced graphene metasurfaces based on simple ribbon geometries to efficiently steer infrared light in the reflective configuration. The graphene ribbons coupled with a subwavelength-thick optical cavity substantially enhance the light-graphene interaction, allowing us to achieve almost 2π phase modulation and high reflectivity amplitude. Anomalous reflection, reflective focusing lenses and non-diffracting beam generation with capability of dynamic tuning have been successfully demonstrated. Although we have focused on the far-infrared frequency, the design strategy can be readily applied to mid-infrared region by varying the dimension of graphene ribbons and thus their plasmonic resonance. Moreover, extending ribbons to other 2D patterns provides additional degrees of freedom in the design, promising other important advantages for light steering such as 3D focusing, polarization-independent functionalities, and strong polarization conversion. Leveraging the remarkable plasmonic properties of graphene at infrared wavelengths, the graphene metasurfaces demonstrated here manifest unprecedented control over the electromagnetic properties of materials on a completely 2D platform, which enable disruptive light-weight, ultra-compact, and high-speed optical architectures for diverse applications.

## Methods

In the terahertz and infrared region, the optical conductivity of graphene is dominated by the intraband transition. It can be described by a semi-classical Drude model^64^,


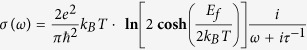


where *k*_*B*_ is the Boltzmann constant, *Τ* is the temperature, *E*_*f*_ is the absolute value of the Fermi energy, and *τ* is the electron relaxation time. When the Fermi energy *E*_*f*_ is much larger than the thermal energy *k*_*B*_*T*, the conductivity of graphene can be further simplified to 
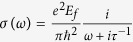
. The electron relaxation time *τ* is related to the carrier mobility *μ*, the Fermi energy *E*_*f*_ and Fermi velocity *v*_*f*_ as *τ* = *μE*_*f*_/*ev*_*f*_^2^, which indicates that increasing carrier mobility of graphene will reduce the loss and improve the device efficiency. The carrier mobility *μ* of graphene film ranges from ∼1000 cm^2^/Vs in chemical vapor deposition (CVD)-grown graphene to 230,000 cm^2^/Vs in suspended exfoliated graphene. In our simulations, we assume the Fermi energy of graphene *E*_*f*_ around 0.64 eV, a moderate mobility *μ* = 10,000 cm^2^/Vs, Fermi velocity *v*_*f*_ = 10^6 ^m/s and scattering time *τ* = 6.4 × 10^−13^ s^27^,^30^. Please note that the Fermi energy as high as *E*_*f*_ = 1–2 eV has been reported^65^,^66^, and graphene mobility at the level of 10,000 cm^2^/Vs has been achieved by using a hexagonal boron nitride substrate even with thickness as small as 14 nm^67^,^68^. The surface conductivity can be converted into a volume conductivity *σ*_*v*_ = *σ*/*t* assuming that *t* is a very small value^31^. Such an approach has been widely used in previous numerical work. However, it imposes significant meshing load and simulation time. In the finite element simulations based on commercial electromagnetic solver COMSOL Multiphysics, we apply transition boundary condition that assigns the conductivity to a single interface instead of a film with finite thickness. Our comparison results confirm that this method is consistent with previous one when the thickness of graphene film approaches to zeros. As a result, we are able to greatly relieve the meshing difficulty, save memory, and shorten the simulation time. An infrared transparent material, such as Potassium Bromide (KBr), Barium Fluoride (BaF_2_), Calcium Fluoride (CaF_2_), or Magnesium Fluoride (MgF_2_), is employed in the design as the dielectric spacing layer, which is assumed to have a refractive index of 1.4.

## Additional Information

**How to cite this article**: Li, Z. *et al.* Graphene Plasmonic Metasurfaces to Steer Infrared Light. *Sci. Rep.* **5**, 12423; doi: 10.1038/srep12423 (2015).

## Supplementary Material

Supplementary Information
