# Supplementary material for: Graphene Plasmonic Metasurfaces to Steer Infrared Light
Source: Sci Rep. 2015 Jul 23;5:12423. doi: 10.1038/srep12423 (PMC5378890; doi:10.1038/srep12423)
Supplement: Supplementary Information [file srep12423-s1.doc]

**Supplementary Information**

**Title: Graphene Plasmonic Metasurfaces to Steer Infrared Light**

Zubin Li^1, 2^, Kan Yao^3^, Fengnian Xia^4^, Sheng Shen^5^, Jianguo Tian^2^ and Yongmin Liu^1,3, *^

^1^ Department of Mechanical and Industrial Engineering, Northeastern University, Boston, Massachusetts 02115, United States

^2^ Key Laboratory of Weak-Light Nonlinear Photonics, Ministry of Education, TEDA Applied Physics Institute & School of Physics, Nankai University, Tianjin 300457, China

^3^ Department of Electrical and Computer Engineering, Northeastern University, Boston, Massachusetts 02115, United States

^4^ Department of Electrical Engineering, Yale University, New Haven, Connecticut 06511, United States

^5^ Department of Mechanical Engineering, Carnegie Mellon University, Pittsburgh, Pennsylvania 15213, United States

* Corresponding Author. E-mail: y.liu@neu.edu

1. **Phase Shift by Graphene Ribbons without Back Reflective Mirror**

We utilize a subwavelength-thick optical cavity, which is composed of patterned graphene nanostructures, a dielectric layer and an optically thick metal film to enhance the light-graphene interaction. With this configuration, the phase of the reflected light can vary almost from -π to π, allowing us to design various metasurface devices in the reflective configuration. If we remove the metal film, the phase change of reflection of graphene ribbons ranges only 0 ~ π at most, as shown in Fig. S1.

Fig. S1. The reflectivity and phase with varied ribbon widths for (a) suspended graphene ribbons without the dielectric substrate and metal mirror, and (b) ribbons with infinite dielectric layer but without metal mirror. The period of ribbons is 3 μm. The frequency of infrared light is 12.32 THz and the Fermi energy of graphene is 0.64 eV. The refractive index of the dielectric layer is 1.4.

1. **The Reflectivity and Phase of Graphene Ribbons for TE Incidence**

In our design, we use graphene ribbons (gratings) to excite the localized surface plasmon resonance, which is coupled with an optical cavity, in order to realize the modulation of phase and amplitude of the reflected beam. It is known that only TM polarized light can excite the localized surface plasmon resonance of grating structures. We simulate the case of TE incidence for comparison. As expected, the reflection and the phase remain almost unchanged with varied ribbon widths as shown in Fig. S2.

Fig. S2. The reflectivity and phase with varied ribbon widths for TE incidence. The geometric parameters are the same with those in Fig. 2(c).

1. **Graphene Metasurfaces for Anomalous Reflection**

In the design we set 36 graphene ribbons with periodicity of 3 μm. The phase shift between two adjacent unit cells is π/18. Then from Fig. 2(c), we choose suitable ribbons that can provide these phase differences. All data are shown in the following table.

| No. | Δphase (rad.) | ribbon width (nm) |
| --- | --- | --- |
| 1 | 0 | 2500 |
| 2 | 0.17453 | 1487 |
| 3 | 0.34907 | 1333 |
| 4 | 0.5236 | 1255 |
| 5 | 0.69813 | 1206 |
| 6 | 0.87266 | 1171 |
| 7 | 1.0472 | 1145 |
| 8 | 1.22173 | 1125 |
| 9 | 1.39626 | 1108 |
| 10 | 1.5708 | 1093 |
| 11 | 1.74533 | 1080 |
| 12 | 1.91986 | 1069 |
| 13 | 2.0944 | 1059 |
| 14 | 2.26893 | 1049 |
| 15 | 2.44346 | 1040 |
| 16 | 2.61799 | 1031 |
| 17 | 2.79253 | 1022 |
| 18 | 2.96706 | 1013 |
| 19 | 3.14159 | 1005 |
| 20 | 3.31613 | 995 |
| 21 | 3.49066 | 986 |
| 22 | 3.66519 | 976 |
| 23 | 3.83972 | 964 |
| 24 | 4.01426 | 952 |
| 25 | 4.18879 | 938 |
| 26 | 4.36332 | 921 |
| 27 | 4.53786 | 901 |
| 28 | 4.71239 | 877 |
| 29 | 4.88692 | 845 |
| 30 | 5.06145 | 800 |
| 31 | 5.23599 | 733 |
| 32 | 5.41052 | 607 |
| 33 | 5.58505 | 300 |
| 34 | 5.75959 | 300 |
| 35 | 5.93412 | 300 |
| 36 | 6.10865 | 300 |

One may notice that the ribbon widths are equal as 300 nm for the last four ribbons. The phase difference of graphene ribbons cannot cover complete 2π range because of the finite loss of graphene, and the maximum is about 5.573 rad. in our design. So for the phase difference larger than this value, we just employ graphene ribbons with width of 300 nm, which can be readily realized in experiment. As seen from Fig. 3, such an approximation has negligible effects.

1. **Graphene Metasurfaces for Focusing**

In our simulation, we use 101 units of graphene ribbons to achieve the focusing effect. From the designed focus length *F* = 100 μm and *x* values (the positions where the centers of every ribbon lie), we calculate the phase differences with respect to that of ribbon at *x* = 0 by Eq. (3). Then from Fig. 2(c), we choose suitable ribbons which can provide these phase differences. For a symmetric setup, we only need to choose 51 ribbons and all these data are shown as follows.

| No. | x (μm) | Δphase (rad.) | ribbon width (nm) |
| --- | --- | --- | --- |
| 1 | 0 | 0 | 2500 |
| 2 | 3 | 0.01161 | 2100 |
| 3 | 6 | 0.0464 | 1806 |
| 4 | 9 | 0.10429 | 1611 |
| 5 | 12 | 0.18512 | 1477 |
| 6 | 15 | 0.28867 | 1374 |
| 7 | 18 | 0.41468 | 1299 |
| 8 | 21 | 0.56282 | 1243 |
| 9 | 24 | 0.73272 | 1198 |
| 10 | 27 | 0.92397 | 1163 |
| 11 | 30 | 1.13612 | 1134 |
| 12 | 33 | 1.36867 | 1110 |
| 13 | 36 | 1.62111 | 1089 |
| 14 | 39 | 1.89288 | 1071 |
| 15 | 42 | 2.18344 | 1054 |
| 16 | 45 | 2.49219 | 1037 |
| 17 | 48 | 2.81856 | 1021 |
| 18 | 51 | 3.16194 | 1004 |
| 19 | 54 | 3.52173 | 984 |
| 20 | 57 | 3.89735 | 960 |
| 21 | 60 | 4.2882 | 929 |
| 22 | 63 | 4.69369 | 880 |
| 23 | 66 | 5.11324 | 784 |
| 24 | 69 | 5.5463 | 353 |
| 25 | 72 | 5.99231 | 300 |
| 26 | 75 | 0.16755 | 1497 |
| 27 | 78 | 0.63786 | 1221 |
| 28 | 81 | 1.11956 | 1136 |
| 29 | 84 | 1.61216 | 1090 |
| 30 | 87 | 2.11518 | 1057 |
| 31 | 90 | 2.62818 | 1031 |
| 32 | 93 | 3.15072 | 1004 |
| 33 | 96 | 3.68237 | 975 |
| 34 | 99 | 4.22275 | 935 |
| 35 | 102 | 4.77146 | 867 |
| 36 | 105 | 5.32814 | 678 |
| 37 | 108 | 5.89244 | 300 |
| 38 | 111 | 0.18084 | 1479 |
| 39 | 114 | 0.75939 | 1193 |
| 40 | 117 | 1.34459 | 1112 |
| 41 | 120 | 1.93617 | 1068 |
| 42 | 123 | 2.53385 | 1035 |
| 43 | 126 | 3.13735 | 1005 |
| 44 | 129 | 3.74643 | 971 |
| 45 | 132 | 4.36086 | 920 |
| 46 | 135 | 4.9804 | 823 |
| 47 | 138 | 5.60484 | 300 |
| 48 | 141 | 6.23397 | 300 |
| 49 | 144 | 0.58441 | 1236 |
| 50 | 147 | 1.22235 | 1124 |
| 51 | 150 | 1.86442 | 1073 |

For the same reason discussed in Sec. 3, the ribbon widths are set as 300 nm for No. 25, 37, 47, and 48, to approximate the required phase that is larger than 5.573 rad.

1. **Graphene Metasurfaces for Exciting Airy Beam**

In our simulation, we use two ribbon widths for every peak and valley shown in Fig. 6(c) respectively. For every peak or valley location, the ribbons are set periodically with period of 3μm. All data are shown in the following table.

| No. | x range (μm) | Δphase (rad.) | width (nm) |
| --- | --- | --- | --- |
| 1 | -54 ~ 60 | 0 | 858 |
| 2 | -96 ~ -54 | π | 1085 |
| 3 | -129 ~ -96 | 0 | 858 |
| 4 | -159 ~ -129 | π | 1085 |
| 5 | -186 ~ -159 | 0 | 858 |
| 6 | -210 ~ -186 | π | 1085 |
| 7 | -234 ~ -210 | 0 | 858 |
| 8 | -258 ~ -234 | π | 1085 |
| 9 | -279 ~ -258 | 0 | 858 |
| 10 | -300 ~ -279 | π | 1085 |
